# Sex Differences in Brain and Cognition in *de novo* Parkinson's Disease

**DOI:** 10.3389/fnagi.2021.791532

**Published:** 2022-01-06

**Authors:** Javier Oltra, Carme Uribe, Anna Campabadal, Anna Inguanzo, Gemma C. Monté-Rubio, Maria J. Martí, Yaroslau Compta, Francesc Valldeoriola, Carme Junque, Barbara Segura

**Affiliations:** ^1^Medical Psychology Unit, Department of Medicine, Institute of Neurosciences, University of Barcelona, Barcelona, Spain; ^2^Institute of Biomedical Research August Pi i Sunyer (IDIBAPS), Barcelona, Spain; ^3^Research Imaging Centre, Centre for Addiction and Mental Health, Campbell Family Mental Health Research Institute, University of Toronto, Toronto, ON, Canada; ^4^Centro de Investigación Biomédica en Red sobre Enfermedades Neurodegenerativas, Hospital Clínic de Barcelona, Barcelona, Spain; ^5^Parkinson's Disease and Movement Disorders Unit, Neurology Service, Hospital Clínic de Barcelona, Institute of Neurosciences, University of Barcelona, Barcelona, Spain

**Keywords:** Parkinson's disease, sex differences, magnetic resonance imaging, gray matter atrophy, cognitive impairment

## Abstract

**Background and Objective:** Brain atrophy and cognitive impairment in neurodegenerative diseases are influenced by sex. We aimed to investigate sex differences in brain atrophy and cognition in *de novo* Parkinson's disease (PD) patients.

**Methods:** Clinical, neuropsychological and T1-weighted MRI data from 205 PD patients (127 males: 78 females) and 69 healthy controls (40 males: 29 females) were obtained from the PPMI dataset.

**Results:** PD males had a greater motor and rapid eye movement sleep behavior disorder symptomatology than PD females. They also showed cortical thinning in postcentral and precentral regions, greater global cortical and subcortical atrophy and smaller volumes in thalamus, caudate, putamen, pallidum, hippocampus, and brainstem, compared with PD females. Healthy controls only showed reduced hippocampal volume in males compared to females. PD males performed worse than PD females in global cognition, immediate verbal recall, and mental processing speed. In both groups males performed worse than females in semantic verbal fluency and delayed verbal recall; as well as females performed worse than males in visuospatial function.

**Conclusions:** Sex effect in brain and cognition is already evident in *de novo* PD not explained by age *per se*, being a relevant factor to consider in clinical and translational research in PD.

## Introduction

Parkinson's disease (PD) has a 2-fold higher incidence in males reported in early population-based studies (Baldereschi et al., 2000). Consistent with previous meta-analytic studies (Wooten et al., 2004; Taylor et al., 2007), the most recent data revealed that the male-female ratio is around 1.50 for prevalence and incidence (Moisan et al., 2016). Moreover, the male sex in PD is associated with earlier disease onset, more severe motor symptoms and progression, and more frequent cognitive decline compared with the female sex (Meoni et al., 2020). Previous literature suggested that the neuroprotective effect of estrogens could be one of the key factors to explain such differences (Meoni et al., 2020).

Neuropsychological studies show that PD males had worse performance than PD females in global cognition (Szewczyk-Krolikowski et al., 2014; Liu et al., 2015; Lin et al., 2018; Bakeberg et al., 2021), memory (Liu et al., 2015; Lin et al., 2018; Bakeberg et al., 2021), verbal fluency (Szewczyk-Krolikowski et al., 2014; Lin et al., 2018; Reekes et al., 2020; Bakeberg et al., 2021), processing speed (Lin et al., 2018; Reekes et al., 2020), and inhibition (Reekes et al., 2020) tasks. By contrast, females have increased impairment in visuospatial functions (Liu et al., 2015; Lin et al., 2018; Bakeberg et al., 2021). A recent meta-analysis highlights that twenty-two studies reported segregated results for males and females regarding executive functions, ten for visuospatial skills, and nine for memory. In this context, significant effect sizes showed more impairment in males for executive functions (Curtis et al., 2019). Moreover, a longitudinal study involving a large sample of PD concluded that females had a lower risk of developing cognitive impairment (Iwaki et al., 2021). Cognitive decline is more pronounced in males (Liu et al., 2017; Bakeberg et al., 2021), and there is an increased rate of progression to mild cognitive impairment (Cholerton et al., 2018; Bakeberg et al., 2021) and dementia in males (Cholerton et al., 2018).

A recent review highlighted the lack of neuroimaging studies centered on sex differences in PD, despite the clinical and epidemiological evidence (Salminen et al., 2021). To our knowledge, there are only two structural magnetic resonance imaging (MRI) studies testing sex differences in gray matter brain atrophy. Yadav et al. reported significant thinning in several cortical regions in males compared to females in treated PD using cortical thickness (CTh) (Yadav et al., 2016). In *de novo* PD patients, Tremblay et al. did not find sex differences in CTh (Tremblay et al., 2020). However, deformed-based morphometry (DBM) analyses showed sex differences in cortical regions in both directions. Males had more atrophy than females in eleven regions whereas females had more atrophy than males in only six regions. Regarding subcortical gray matter atrophy by DBM, they found more atrophy in males than females in the left thalamus. Thus, the authors concluded that males with *de novo* PD overall had more regional atrophy than females, mainly in cortical regions. In addition, both mentioned works found male-specific structural connectivity disruptions in PD (Yadav et al., 2016; Tremblay et al., 2020).

In this study, our main objective is to analyze sex differences in brain atrophy in a large sample of newly diagnosed drug-naïve PD patients, *de novo* PD patients. We used, for the first time, with that purpose global and subcortical volumetry, as well as cortical thickness analyses. We also analyzed sex differences in neuropsychological performance.

## Methods

### Participants

Two hundred and five *de novo* PD patients and 69 healthy controls from the Parkinson's Progression Markers Initiative database (PPMI, for up-to-date information of the study visit http://www.ppmi-info.org) (Marek et al., 2011), classified by sex: 127 *de novo* PD males, 78 *de novo* PD females, 40 control males, and 29 control females. All participating PPMI sites received approval from an ethical standards committee and obtained written informed consent from all participants in the study.

Inclusion criteria for PD were: (a) recent PD diagnosis with asymmetric resting tremor or asymmetric bradykinesia, or two from among bradykinesia, resting tremor, and rigidity; (b) absence of levodopa intake; (c) DaTSCAN evidence of significant dopamine transporter deficit consistent with PD diagnosis. Inclusion criteria for both groups were: (d) T1-weighted images available; and (e) age older than 50 and younger than 85 years old. Exclusion criteria were: (a) diagnosis of dementia; (b) significant psychiatric, neurologic, or systemic comorbidity; (c) a first-degree family member with PD; and (d) presence of MRI motion artifacts, field distortions, intensity inhomogeneities, or detectable structural brain lesions. The flow diagram of sample selection is shown in Supplementary Figure 1, see Supplementary Methods 1 to comorbidity exclusion reasons after MRI preprocessing.

### Clinical and Neuropsychological Assessments

Clinical assessment included disease severity measured by the Movement Disorders Society Unified PD Rating Scale (MDS-UPDRS) and motor severity by the MDS-UPDRS motor section (MDS-UPDRS Part III), disease stage by Hoehn and Yahr scale (H&Y), general cognition by Montreal Cognitive Assessment (MoCA), and rapid eye movement sleep behavior disorder (RBD) symptomatology by the REM Sleep Behavior Disorder Screening Questionnaire (RBDSQ) (Marek et al., 2011). Neuropsychological battery included: phonemic (letter “f”) and semantic (animals, fruits and vegetables) verbal fluency; Symbol Digit Modalities Test (SDMT); Letter-Number Sequencing (LNS); Benton Judgment of Line Orientation 15-item short form (JLO); and Hopkins Verbal Learning Test-Revised (HVLT-R) (Marek et al., 2011). Neuropsychological measures were z-scored calculated based on the control group's means and standard deviations.

### MRI Images

T1-weighted scans were acquired using 1.5 or 3-Tesla scanners using magnetization prepared rapid gradient-echo imaging (MPRAGE) sequences. Typical parameters were repetition time = 5–11 ms; echo time = 2–6 ms; slice thickness 1–1.5 mm; inter-slice gap 0 mm; voxel size 1 × 1 × 1.2 mm; matrix 256 × 160 minimum. There were no differences in the distribution of 1.5 and 3-Tesla images across groups (Supplementary Table 1).

CTh, subcortical and cortical volumes were estimated using the automated processing stream and specific segmentation tools of FreeSurfer (version 6.0, https://surfer.nmr.mgh.harvard.edu). The main preprocessing procedures are removal of non-brain data, intensity normalization (Fischl et al., 2001), tessellation of the gray matter (GM)/white matter (WM) boundary, automated topology correction (Dale et al., 1999; Ségonne et al., 2007), accurate surface deformation to identify tissue borders (Dale and Sereno, 1993; Fischl and Dale, 2000; Fischl et al., 2002), cortical thickness calculation as the distance between the WM and GM surfaces at each vertex of the reconstructed cortical mantle (Fischl et al., 2002). After preprocessing and quality control (check the accuracy of registration, skull stripping, segmentation, and cortical surface reconstruction), errors were fixed by automated and manual interventions following standard procedures and were discarded when correction was not possible. The smoothing of the maps of CTh was fixed at full width half maximum (FWHM) of 15 mm of a circularly symmetric Gaussian kernel across the surface. Global average thickness for both hemispheres was calculated ([lh thickness^*^lh surface area] + [rh thickness^*^rh surface area]/[lh surface area + rh surface area]).

The used atlas for volumetry corresponds to the Automatic Subcortical Segmentation Atlas (Aseg Atlas) (Fischl et al., 2002). Deep gray GM mean volumes, estimated total intracranial volume (eTIV), total cortical and subcortical GM were also estimated (Fischl et al., 2002). GM volumes were bilateralized [(left volume + right volume)/2] and transformed to ratios in percentages [(volume/eTIV)^*^100].

### Statistical Analyses

The main effects of group and sex were computed for sociodemographic variables by two-way analysis of variance (ANOVA) applying Bonferroni correction for quantitative measures to *post-hoc* tests. The main effect of sex, the within-group effect of sex and the group-by-sex interaction were computed for clinical, neuropsychological, and MRI volumetry measures by two-way analyses of covariance (ANCOVA), Bonferroni correction was applied to *post-hoc* tests and partial eta squared was computed. Pearson's chi-squared tests were used to compute differences in categorical measures. Differences in age of onset and disease duration were computed by *t*-test. Analyses were performed with IBM SPSS Statistics 27.0.0 (2020; IBM Corp., Armonk, NY).

Inter-group whole-brain CTh comparisons were performed in FreeSurfer v6.0 using a vertex-by-vertex general linear model; including CTh as a dependent factor, group as an independent factor, and demeaned age and years of education as covariates. All results were corrected for multiple comparisons using a pre-cached cluster-wise Monte Carlo simulation with 10,000 iterations.

For all analyses, the statistical significance threshold was set at *p* < 0.05.

## Results

### Clinical Characteristics

Males were significantly older than females in the PD and healthy control groups, as well that the control group had more years of education than the PD group (Table 1). Subsequent analyses included age and years of education as covariates as required.

**Table 1:** Demographic and clinical characteristics of PD and HC females and males.

		**PD**	**HC**	**Sex main effect test stat (*P*-value)**	**Group main effect F stat (*P*-value)**
Age, y	F	61.76 (7.50)	60.55 (5.86)	7.471 (0.007)^a^, ^b^	0.223 (0.637)
	M	63.80 (7.24)	64.05 (7.11)		
Education, y	F	15.36 (2.96)	16.24 (2.86)	2.588 (0.109)	5.820 (0.017)
	M	15.91 (2.96)	17.00 (2.48)		
Age of onset, y	F	60.81 (7.52)		1.947 (0.053)	
	M	62.84 (7.10)			
Disease duration, m	F	10.50 (7.93)		0.310 (0.757)	
	M	10.82 (6.64)			
MDS-UPDRS	F	29.58 (10.69)		3.369 (0.068)	
	M	33.17 (13.04)			
Part III	F	18.62 (7.56)		5.510 (0.020)	
	M	21.62 (8.86)			
H&Y, *n*, 1/2/3	F	32/45/1		0.284 (0.867)	
	M	56/70/1			
RBDSQ	F	3.73 (1.99)	1.52 (1.21)	2.557 (0.111)^a^	58.211 (<0.001)
	M	4.53 (2.73)	1.88 (1.40)		

*Data are presented by groups as mean (SD), except for H&Y. Two-way analyses of variance (ANOVA) with post-hoc tests corrected by Bonferroni were used for all demographic variables. Two-way analyses of covariance (ANCOVA) with age as a covariate with post-hoc tests corrected by Bonferroni were used for all clinical variables. Except for age of onset and PD duration, by two-sample t-test and H&Y, by Pearson's chi-squared*.

a *Sex differences in PD group (p < 0.05)*.

b *Sex differences in HC group (p < 0.05)*.

*F, female; H&Y, Hoehn and Yahr scale; HC, healthy control; m, months; M, male; MDS-UPDRS, Movement Disorder Society Unified Parkinson's Disease Rating Scale; PD, Parkinson's disease; RBDSQ, REM Sleep Behavior Disorder Screening Questionnaire; y, years*.

A significant sex effect was found in motor severity (MDS-UPDRS Part III) in the PD group. Despite similar disease duration, males had more severe motor symptoms than females. Moreover, *post-hoc* tests showed that in the PD group, males had more RBD symptoms (RBDSQ) than females (Table 1).

### Neuropsychological Performance

**Figure 1:**
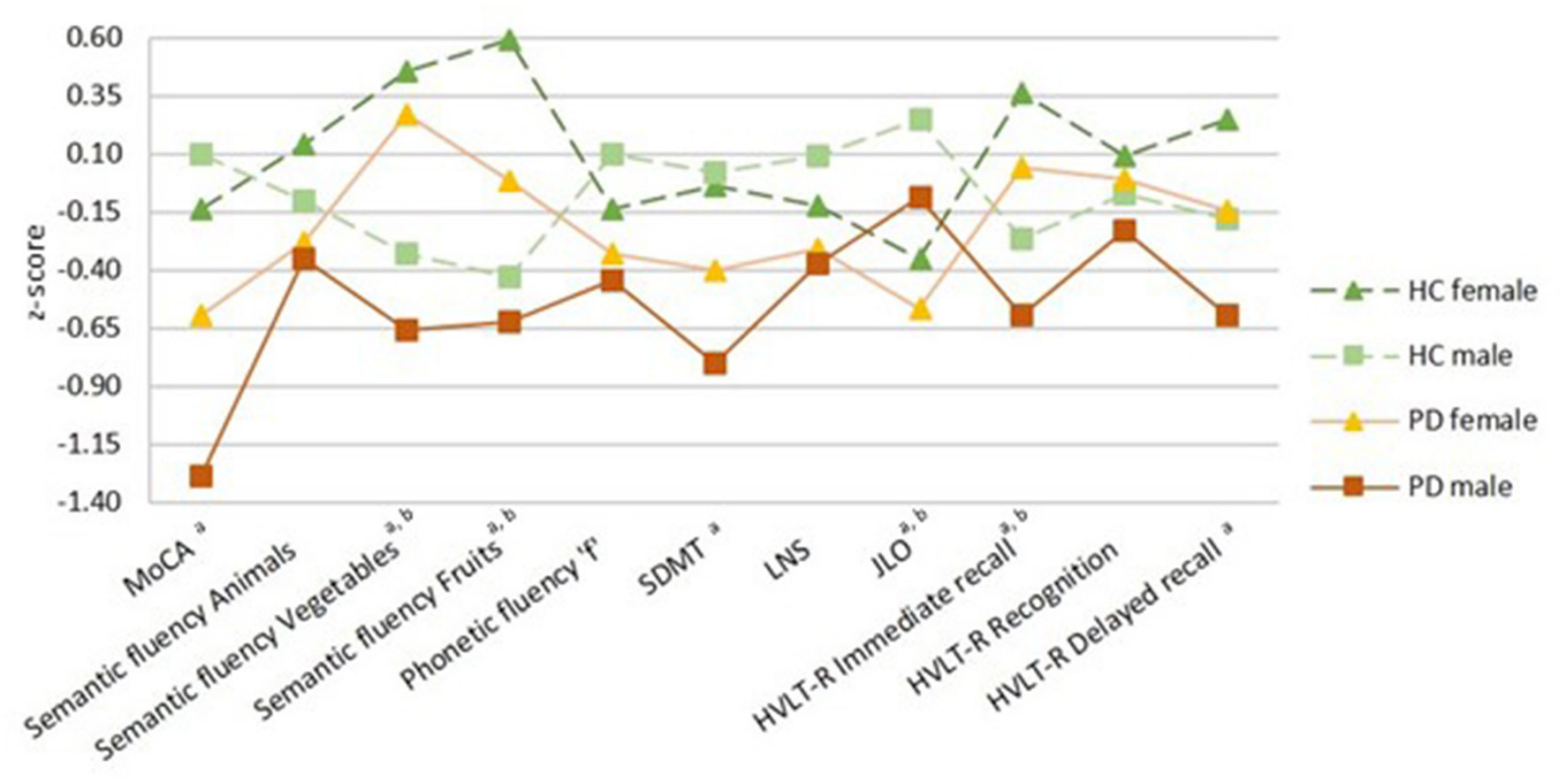
Neuropsychological performance. Healthy controls (HC) groups in green, Parkinson's disease (PD) groups in warm colors; darker for females and lighter for males. Females represented by filled triangles and males by filled squares. PD by a discontinuous line and HC by a continuous line. Data are presented as z-scores. Lower z-scores indicate worse performance. Two-way analyses of covariance (ANCOVA) with age and education as covariates with *post-hoc* tests corrected by Bonferroni were used for all variables. MoCA, Montreal Cognitive Assessment; SDMT, Symbol Digit Modalities Test; LNS, Letter-Number Sequencing; JLO, Benton Judgment of Line Orientation; HVLT-R, Hopkins Verbal Learning Test-Revised. ^a^Sex differences in PD group, ^b^sex differences in HC group (*p* < 0.05).

There was a significant sex effect in semantic fluency, JLO, and HVLT-R delayed recall. A significant group-by-sex interaction was found in MoCA (*F* = 4.215, *p* = 0.041, ηp2 = 0.015). In the PD group, *post-hoc* tests revealed that males performed worse than females in MoCA, semantic fluency, SDMT, and HVLT-R immediate and delayed recall. As well, in the healthy control group, males performed lower than females in semantic fluency and HVLT-R immediate recall. In both groups, females had lower scores than males in the JLO (Figure 1; Table 2; Supplementary Table 2).

**Table 2:** Neuropsychological performance of PD and HC females and males.

		**PD**		**HC**		**Sex main effect F stat (*P*-value)**	**Partial eta squared**
		**Mean (SD)**	**Median (IQR)**	**Mean (SD)**	**Median (IQR)**		
MoCA	F	−0.559 (1.742)	−0.078 (2.62)	−0.139 (0.900)	−0.078 (1.74)	1.047 (0.307)^a^	0.004
	M	−1.287 (1.884)	−0.950 (2.62)	0.096 (1.067)	−0.078 (1.74)		
Semantic fluency
Animals	F	−0.278 (0.885)	−0.490 (1.11)	0.141 (0.970)	0.065 (1.11)	1.022 (0.313)	0.004
	M	−0.351 (0.951)	−0.490 (1.29)	−0.102 (1.021)	−0.305 (1.11)		
Vegetables	F	0.271 (1.098)	0.157 (1.36)	0.457 (1.031)	0.700 (1.36)	32.796 (<0.001)^a^, ^b^	0.110
	M	−0.658 (1.052)	−0.927 (1.63)	−0.331 (0.845)	−0.385 (1.29)		
Fruits	F	−0.018 (0.901)	−0.123 (1.20)	0.592 (0.806)	0.600 (0.96)	37.032 (<0.001)^a^, ^b^	0.122
	M	−0.624 (0.962)	−0.846 (1.45)	−0.430 (0.910)	−0.605 (1.20)		
Phonetic fluency “f”	F	−0.308 (0.975)	−0.404 (1.10)	−0.134 (0.916)	−0.294 (1.44)	0.002 (0.963)	0.000
	M	−0.440 (1.009)	−0.515 (1.33)	0.098 (1.057)	−0.073 (1.05)		
SDMT	F	−0.403 (0.934)	−0.348 (1.23)	−0.034 (0.857)	−0.292 (1.12)	0.543 (0.462)^a^	0.002
	M	−0.799 (1.020)	−0.623 (1.34)	0.024 (1.102)	0.100 (1.37)		
LNS	F	−0.307 (0.922)	−0.124 (1.16)	−0.124 (0.907)	−0.124 (1.55)	0.898 (0.344)	0.003
	M	−0.370 (1.042)	−0.510 (1.16)	0.089 (1.065)	−0.124 (1.45)		
JLO	F	−0.564 (1.176)	−0.187 (1.75)	−0.348 (1.125)	−0.187 (1.17)	12.665 (<0.001)^a^, ^b^	0.045
	M	−0.086 (1.164)	0.397 (1.75)	0.251 (0.824)	0.397 (1.17)		
HVLT-R
Immediate recall	F	0.046 (0.986)	0.027 (1.14)	0.366 (0.897)	0.485 (1.26)	2.916 (0.089)^a^, ^b^	0.059
	M	−0.594 (1.148)	−0.430 (1.60)	−0.264 (0.997)	−0.423 (1.54)		
Recognition	F	−0.010 (0.854)	0.340 (0.93)	0.095 (0.879)	0.340 (0.62)	1.632 (0.203)	0.006
	M	−0.230 (0.940)	0.031 (0.62)	−0.070 (1.085)	0.031 (0.85)		
Delayed recall	F	−0.144 (0.935)	−0.078 (1.55)	0.249 (0.768)	0.335 (0.83)	10.240 (0.002)^a^	0.037
	M	−0.596 (1.078)	−0.492 (1.65)	−0.182 (1.114)	−0.078 (1.65)		

*Data are presented in z-scores. Two-way analyses of covariance (ANCOVA) with age and education as covariates with post-hoc tests corrected by Bonferroni were used for all variables*.

a *Sex differences in PD group (p < 0.05)*.

b *Sex differences in HC group (p < 0.05)*.

*F, female; HC, healthy control; HVLT-R, Hopkins Verbal Learning Test-Revised; JLO, Benton Judgment of Line Orientation; LNS, Letter-Number Sequencing; M, male; MoCA, Montreal Cognitive Assessment; PD, Parkinson's disease; SDMT, Symbol Digit Modalities Test*.

### MRI-Derived Measures

There was a significant effect of sex in global GM volumes, *post-hoc* tests revealed that in the PD group males had smaller total cortical and subcortical GM volumes than females. Regarding subcortical volumetry, a significant main effect of sex was found in the bilateral thalamus, caudate, putamen, and hippocampus. *Post-hoc* tests showed that in the PD group, males had smaller volumes than females in the bilateral thalamus, caudate, putamen, pallidum, hippocampus, and brainstem. Within the healthy control group, males had smaller bilateral volume than females in the hippocampus (Table 3; Supplementary Table 3).

**Table 3:** MRI-derived measures of between sex comparisons of PD and HC females and males.

		**PD**		**HC**		**Sex main effect F stat (*P*-value)**	**Partial eta squared**
		**Mean (SD)**	**Median (IQR)**	**Mean (SD)**	**Median (IQR)**		
**Global atrophy**
Cortical	F	29.598 (2.266)	29.360 (2.07)	30.114 (1.800)	30.091 (2.46)	8.721 (0.003)^a^	0.032
	M	28.149 (2.153)	28.309 (2.29)	29.387 (2.256)	29.246 (2.42)		
Subcortical	F	3.642 (0.282)	3.603 (0.39)	3.686 (0.270)	3.640 (0.38)	12.188 (<0.001)^a^	0.043
	M	3.467 (0.244)	3.452 (0.33)	3.544 (0.280)	3.496 (0.25)		
Mean CTh	F	2.415 (0.095)	2.426 (0.11)	2.436 (0.102)	2.416 (0.10)	1.051 (0.306)	0.004
	M	2.389 (0.119)	2.415 (0.14)	2.411 (0.124)	2.412 (0.14)		
**Deep GM nuclei**
Thalamus	F	0.462 (0.043)	0.468 (0.06)	0.460 (0.030)	0.457 (0.04)	7.874 (0.005)^a^	0.029
	M	0.436 (0.039)	0.434 (0.05)	0.443 (0.046)	0.443 (0.07)		
Caudate	F	0.224 (0.026)	0.219 (0.03)	0.222 (0.028)	0.219 (0.03)	7.948 (0.005)^a^	0.029
	M	0.210 (0.025)	0.208 (0.03)	0.215 (0.024)	0.211 (0.03)		
Putamen	F	0.295 (0.036)	0.291 (0.05)	0.303 (0.038)	0.300 (0.06)	5.690 (0.018)^a^	0.021
	M	0.281 (0.032)	0.282 (0.04)	0.290 (0.034)	0.284 (0.04)		
Pallidum	F	0.128 (0.015)	0.127 (0.02)	0.126 (0.013)	0.126 (0.02)	2.275 (0.133)^a^	0.008
	M	0.124 (0.014)	0.123 (0.02)	0.124 (0.015)	0.121 (0.02)		
Hippocampus	F	0.269 (0.030)	0.268 (0.04)	0.279 (0.028)	0.283 (0.04)	18.927 (<0.001)^a^, ^b^	0.066
	M	0.250 (0.027)	0.247 (0.04)	0.257 (0.026)	0.252 (0.03)		
Accumbens	F	0.032 (0.007)	0.031 (0.01)	0.032 (0.006)	0.032 (0.01)	1.601 (0.207)	0.006
	M	0.030 (0.006)	0.029 (0.01)	0.031 (0.004)	0.030 (0.01)		
Amygdala	F	0.105 (0.017)	0.104 (0.02)	0.110 (0.013)	0.111 (0.02)	0.028 (0.868)	0.000
	M	0.104 (0.013)	0.103 (0.02)	0.110 (0.014)	0.108 (0.01)		
Brainstem	F	1.412 (0.122)	1.391 (0.16)	1.382 (0.100)	1.384 (0.13)	0.662 (0.417)^a^	0.002
	M	1.359 (0.120)	1.365 (0.18)	1.390 (0.134)	1.392 (0.20)		

Volumetric variables are presented in ratios as percentages estimated by [(volume/eTIV) * 100]. Two-way analyses of covariance (ANCOVA) with age and education as covariates with post-hoc tests corrected by Bonferroni were used for all variables.

a *Sex differences in PD group*.

b *Sex differences in HC group (p < 0.05)*.

*CTh, cortical thickness; F, female; GM, gray matter; HC, healthy control; M, male; PD, Parkinson's disease*.

**Figure 2:**
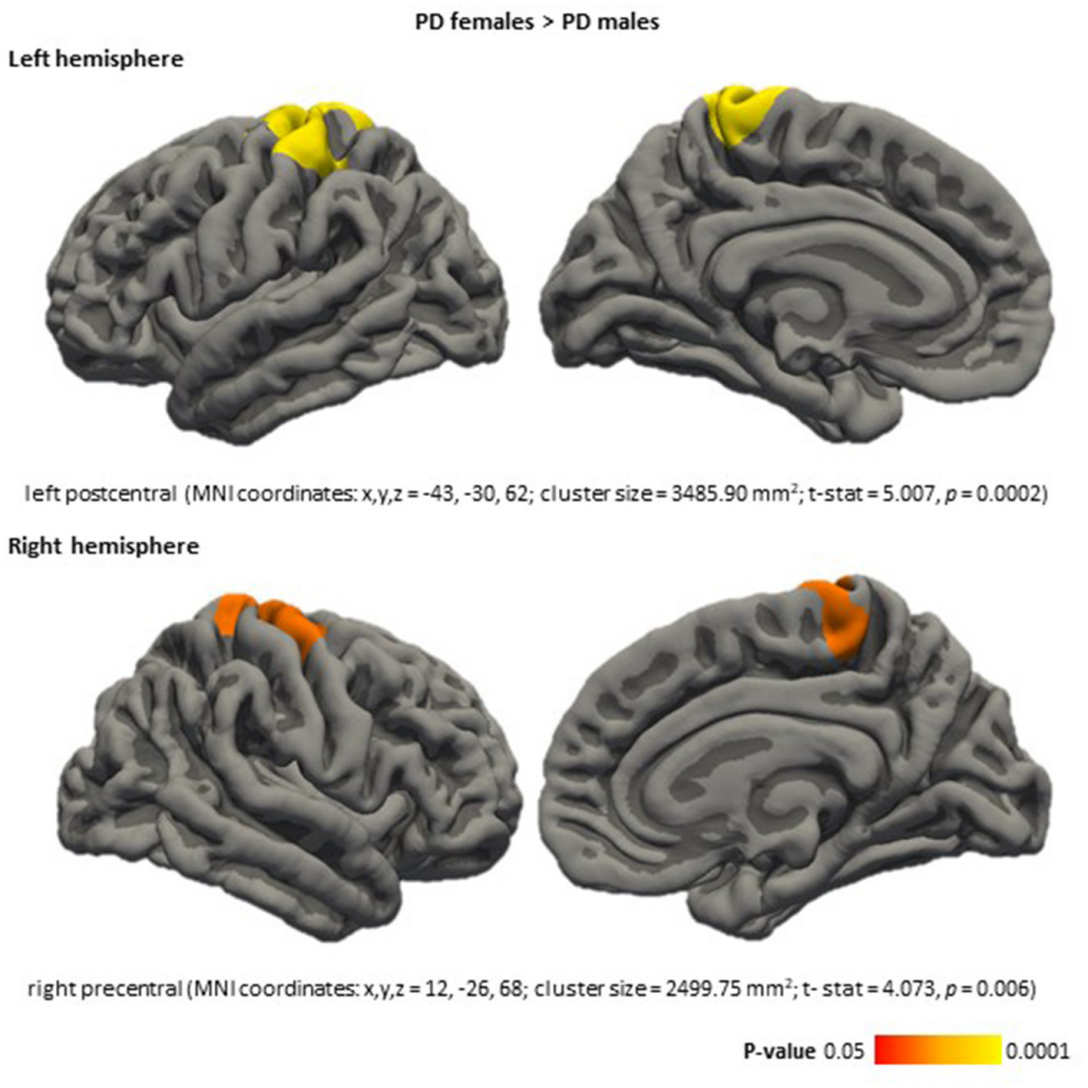
Cortical thickness differences between PD females and PD males, in the PD females > PD males direction. Color maps indicate clusters with significant differences (corrected *p* < 0.05). Results were corrected by Monte Carlo simulation. Detailed information is included for each significant cluster: cortical area, MNI coordinates (x, y, z), cluster size (mm^2^), test stat (*t*-stat), and *P*-value (*p*). PD, Parkinson's disease.

Vertex-wise analyses revealed sex effects in cortical thickness in the PD group, males had thinning in left postcentral (MNI coordinates: x, y, z = −43, −30, 62; cluster size = 3,485.90 mm^2^; *t*-stat = 5.007, *p* < 0.001) and right precentral (MNI coordinates: x, y, z = 12, −26, 68; cluster size = 2,499.75 mm^2^; *t*-stat = 4.0728, *p* = 0.006) compared with females (Figure 2).

## Discussion

Our results point to a more severe clinical, cognitive, and neurodegenerative profile in *de novo* PD males compared with *de novo* PD females, despite similar disease duration and adjusting the results by age and education. Clinically, PD males had increased motor severity (MDS-UPSRS Part III) than PD females. This result is in keeping with increased cortical thinning in cortical motor region, as well as increased volume reductions in the bilateral thalamus and basal ganglia structures such as putamen, pallidum, and caudate after controlling by eTIV.

There is only one similar study performed with a *de novo* PD sample investigating the brain differences between sexes (Tremblay et al., 2020). In that study, the authors did not find sex-related differences in CTh and found larger volume in PD females than PD males in the left thalamus by DBM means. In the PD group, our results showed larger subcortical gray matter volume in females than in males in the bilateral thalamus. This result partially agrees with the mentioned result that showed reduced left thalamus volume in males compared with females. Remarkably, different atlases were used to define subcortical structures. Furthermore, the differences in the CTh results between both studies could be explained by differences in the estimation pipelines (Masouleh et al., 2020), and the statistical analysis software employed, as well as the MRI analytical approaches based on CTh atlas-based parcellations or whole-brain vertex-wise CTh maps.

We found cortical thinning in *de novo* PD males compared with *de novo* PD females in the left postcentral and right precentral areas. A previous study including treated PD patients with larger disease duration (between 2.13 and 3.69 years) reported cortical thinning in PD males in the left precentral and right postcentral areas compared with PD females. As well, significant thinning in temporal and occipital regions in PD males compared with PD females (Yadav et al., 2016). These results might suggest sex differences in brain atrophy associate with the illness progression. However, longitudinal MRI studies are required.

Adult males have larger volumes than females in some subcortical gray matter structures, such as the nuclei accumbens, according to a study performed in a sample of 5,216 participants with an age range between 44 and 77 years (Ritchie et al., 2018); as well as, the amygdala, hippocampus, and putamen, according to other study performed in a sample of 2,838 participants with and age range between 21 and 90 years (Lotze et al., 2019), both controlling for age and total brain volume. In our study, sex differences in PD could be attributed to the neurodegenerative process rather than normal aging because, in healthy controls, we only found sex differences in the hippocampus. Nevertheless, it is noteworthy that the sample used in our study is modest in comparison to previous population-based studies reporting subcortical volumetric differences in healthy subjects. The pattern of atrophy in PD that we have found showed that males have reduced volumes of subcortical nuclei compared with females, thus is the reversed pattern seen in general adult population suggesting a more marked degeneration in males or protective effect of female sex. In this regard, dysregulated gene expression and sex hormones might explain sex differences in PD. Vulnerability in the dopaminergic system, neuroinflammatory cells, and oxidative stress has been suggested as mechanisms that influence sex differences in PD (Cerri et al., 2019).

The neuropsychological results are also in agreement with greater global atrophy in males. Cognitive results showed that PD males had worse performance than PD females in general cognition (MoCA), processing speed (SDMT), and verbal memory (HVLT-R delayed recall). These results agree with previous findings in *de novo* PD showing more impairment in males than females in general cognition (Szewczyk-Krolikowski et al., 2014; Liu et al., 2015; Lin et al., 2018), verbal memory (Liu et al., 2015; Lin et al., 2018), and processing speed (Lin et al., 2018). We obtained sex differences in visuospatial function, in which females performed worse than males in PD and control groups. This result is consistent with previous findings in *de novo* PD (Liu et al., 2015; Lin et al., 2018), and it would reflect premorbid abilities. Greater abilities in line orientation in males were observed in a study performed with 201,000 participants, involving 53 nations (Lippa et al., 2010). This sex differences in visuospatial function also remained in normal aging (Munro et al., 2012; McCarrey et al., 2016).

Our results show modest effect sizes of the main effect of sex in MRI-derived and cognitive measures in the PD group. The interpretation of the data should be made cautiously. Future research needs to consider the role of other co-factors such as environmental and lifestyle variables that could influence brain atrophy and functional outcomes in PD together with biological sex. In this context, diet quality and physical activity have shown a protective effect against the development of PD (Yang et al., 2015; Liu et al., 2021), and MIND and Mediterranean diets has been related to later PD onset, mainly in females (Metcalfe-Roach et al., 2021). Moreover, physical activity interventions have shown improvement in functional outcomes in PD patients (Sharp and Hewitt, 2014). Another relevant factor to consider in further studies is sex differences in modifiable vascular risk factors highly related to lifestyle variables. In this regard, hypertension has been related longitudinally to the development of MCI in PD (Nicoletti et al., 2021).

PPMI study includes multisite data including 1.5 and 3-Tesla MRI acquisitions, therefore field strength differences could be considered a potential confounder in our analyses. In this regard, we checked 1.5 and 3-Tesla acquisitions were equally distributed between our study groups.

Finally, regarding clinical variables, it must be considered that the PD diagnosis in women can be delayed, and age of onset would be biased. However, more evidence is needed concerning the expected time from disease onset to visit with a movement disorder specialist (Saunders-Pullman et al., 2011).

Of interest, other neurodegenerative diseases show relevant differential characteristics between sexes in cognition and brain atrophy. Alzheimer's disease is the most studied among all. Remarkably, females with Alzheimer's disease have higher brain atrophy rates than males (Hua et al., 2010; Ardekani et al., 2016) and have a worse performance in verbal memory tasks compared with males (Chapman et al., 2011; Benke et al., 2013). Thus, consider the effect of sex in neurodegenerative diseases in translational research and clinical trials is a key point in the era of precession medicine.

In conclusion, PD might aggravate the sex differences in cognition and brain atrophy associated with normal aging. The characterization of phenotypic sex differences in Parkinson's disease could be crucial to develop personalized medicine approaches from the early stages of the disease.

## Data Availability Statement

Publicly available datasets were analyzed in this study. This data can be found at: http://www.ppmi-info.org.

## Ethics Statement

All participating PPMI sites received approval from an ethical standards committee and obtained written informed consent from all participants in the study. The patients/participants provided their written informed consent to participate in this study.

## Author Contributions

Research project conception and acquisition of data are explained in Marek et al. (2011). BS and CJ contributed to the design of the study. JO and BS contributed to the analysis of the data. JO, CU, AC, AI, GM-R, CJ, and BS contributed to the interpretation of the data. JO, CU, CJ, and BS contributed to the draft of the article. JO, CU, MJM, YC, FV, CJ, and BS revised the manuscript critically for important intellectual content and approved the final version of the manuscript. All authors contributed to the article and approved the submitted version.

## Funding

This study was sponsored by the Spanish Ministry of Economy and Competitiveness (PSI2017-86930-P), cofinanced by Agencia Estatal de Investigación (AEI), European Regional Development Fund (ERDF) and PID2020-114640GB-I00/MCIN/AEI/10.13039/501100011033, by Generalitat de Catalunya (2017SGR748), and supported by María de Maeztu Unit of Excellence (Institute of Neurosciences, University of Barcelona) MDM-2017-0729, Ministry of Science, Innovation and Universities. JO was supported by a 2018 fellowship from the Spanish Ministry of Science, Universities and Research and co-financed by the European Social Fund (PRE2018-086675). CU was supported by the European Union's Horizon 2020 research and innovation programme under the Marie Sklodowska-Curie fellowship (Grant Agreement 888692). AI was supported by APIF predoctoral fellowship from the University of Barcelona (2017–2018). MJM received grants from Michael J. Fox Foundation for Parkinson's Disease (MJFF): MJF_PPMI_10_001, PI044024.

## Conflict of Interest

MJM received honoraria for advice and lecture from Abbvie, Bial, and Merzt Pharma and grants from Michael J. Fox Foundation for Parkinson's Research (MJFF): MJF_PPMI_10_001, PI044024. YC has received funding in the past 5 years from Union Chimique Belge (UCB pharma), Teva, Medtronic, Abbvie, Novartis, Merz, Piramal Imaging, Esteve, Bial, and Zambon and currently an associate editor for Parkinsonism & Related Disorders. The remaining authors declare that the research was conducted in the absence of any commercial or financial relationships that could be construed as a potential conflict of interest.

## Publisher's Note

All claims expressed in this article are solely those of the authors and do not necessarily represent those of their affiliated organizations, or those of the publisher, the editors and the reviewers. Any product that may be evaluated in this article, or claim that may be made by its manufacturer, is not guaranteed or endorsed by the publisher.
