# Supplementary material for: Sex Differences in Brain and Cognition in de novo Parkinson's Disease
Source: Front Aging Neurosci. 2022 Jan 6;13:791532. doi: 10.3389/fnagi.2021.791532 (PMC8770804; doi:10.3389/fnagi.2021.791532)
Supplement: Supplementary file 1 [file Data_Sheet_1.docx]

Supplementary Material

**Supplementary Table 1**

MRI field strength distribution of the groups

|  | **1.5 T** | **3 T** | **Test stat**  **(*P*-value)** |
| --- | --- | --- | --- |
| HC female | 8 (27.6%) | 21 (72.4%) | 3.213 (0.360) |
| HC male | 6 (15%) | 34 (85%) |  |
| PD female | 20 (25.6%) | 58 (74.5%) |  |
| PD male | 37 (29.1%) | 90 (70.9%) |  |

Data are presented by groups as *n* (%). Pearson's chi-squared was used.

Abbreviations: HC = healthy control; PD = Parkinson’s disease; T = Tesla.

**Supplementary Table 2**

Post hoc tests corresponding to the within-group sex main effect in neuropsychological tasks

|  |  | **Sex main effect**  **F stat (*P*-value)** | **Partial eta squared** |
| --- | --- | --- | --- |
| MoCA | PD | 9.104 (0.003) | 0.033 |
|  | HC | 0.341 (0.560) | 0.001 |
| Semantic fluency |  |  |  |
| Animals | PD | 0.215 (0.644) | 0.001 |
|  | HC | 0.829 (0.363) | 0.003 |
| Vegetables | PD | 38.546 (<0.001) | 0.127 |
|  | HC | 9.142 (0.003) | 0.033 |
| Fruits | PD | 20.412 (<0.001) | 0.071 |
|  | HC | 19.817 (<0.001) | 0.069 |
| Phonetic fluency 'f' | PD | 1.372 (0.242) | 0.005 |
|  | HC | 0.556 (0.457) | 0.001 |
| SDMT | PD | 6.467 (0.012) | 0.024 |
|  | HC | 0.422 (0.516) | 0.002 |
| LNS | PD | 0.025 (0.874) | 0.000 |
|  | HC | 1.456 (0.229) | 0.005 |
| JLO | PD | 9.246 (0.003) | 0.034 |
|  | HC | 5.598 (0.019) | 0.021 |
| HVLT-R |  |  |  |
| Immediate recall | PD | 17.101 (<0.001) | 0.060 |
|  | HC | 5.465 (0.020) | 0.020 |
| Recognition | PD | 2.212 (0.138) | 0.008 |
|  | HC | 0.376 (0.540) | 0.001 |
| Delayed recall | PD | 10.674 (0.001) | 0.039 |
|  | HC | 3.276 (0.071) | 0.012 |

Two-way analyses of covariance (ANCOVA) with age and education as covariates with post-hoc tests corrected by Bonferroni were used for all variables.

Abbreviations: HC = healthy control; HVLT-R = Hopkins Verbal Learning Test-Revised; JLO = Benton Judgment of Line Orientation; LNS = Letter-Number Sequencing; MoCA = Montreal Cognitive Assessment; PD= Parkinson’s disease; SDMT = Symbol Digit Modalities Test.

**Supplementary Table 3**

Post hoc tests corresponding to the within-group sex main effect in MRI derived measures

|  |  | **Sex main effect Test stat (*P*-value)** | **Partial eta squared** |
| --- | --- | --- | --- |
| **Global atrophy** |  |  |  |
| Cortical | PD | 17.997 (<0.001) | 0.063 |
|  | HC | 0.907 (0.342) | 0.003 |
| Subcortical | PD | 16.926 (<0.001) | 0.059 |
|  | HC | 2.748 (0.099) | 0.010 |
| Mean CTh | PD | 0.242 (0.624) | 0.001 |
|  | HC | 1.441 (0.231) | 0.005 |
| **Deep GM nuclei** |  |  |  |
| Thalamus | PD | 15.498 (<0.001) | 0.055 |
|  | HC | 0.923 (0.338) | 0.003 |
| Caudate | PD | 14.422 (<0.001) | 0.051 |
|  | HC | 1.120 (0.291) | 0.004 |
| Putamen | PD | 6.112 (0.014) | 0.022 |
|  | HC | 1.777 (0.184) | 0.007 |
| Pallidum | PD | 4.702 (0.031) | 0.017 |
|  | HC | 0.236 (0.628) | 0.001 |
| Hippocampus | PD | 17.472 (<0.001) | 0.061 |
|  | HC | 6.891 (0.009) | 0.025 |
| Accumbens | PD | 2.802 (0.095) | 0.010 |
|  | HC | 0.243 (0.622) | 0.001 |
| Amygdala | PD | 0.005 (0.941) | 0.000 |
|  | HC | 0.057 (0.812) | 0.000 |
| Brainstem | PD | 7.312 (0.007) | 0.027 |
|  | HC | 0.415 (0.520) | 0.002 |

Two-way analyses of covariance (ANCOVA) with age and education as covariates with post-hoc tests corrected by Bonferroni were used for all variables. Abbreviations: CTh = cortical thickness;  GM = gray matter; HC = healthy control; PD = Parkinson’s disease.

**Supplementary Figure 1.** Flow diagram of sample selection. Abbreviations: FS = FreeSurfer; HC = healthy control; PD = Parkinson’s disease


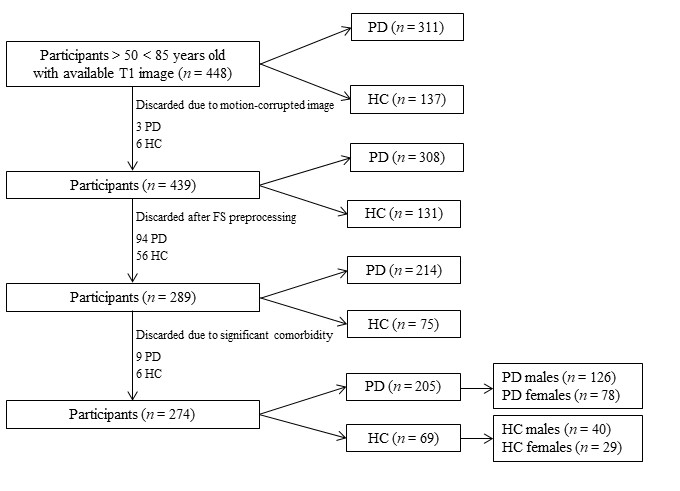


**Supplementary Methods 1**

Clinical and medication databases were checked after MRI preprocessing to make sure that any PD or control participant with relevant comorbidities did not enter in posterior analyses. We excluded 9 PD participants (1 due to seizure and obsessive-compulsive disorder; 1 to depression and attention hyperactivity deficit disorder; 1 to mood disorders, sleep apnea, thymus removal an thalamus removal; 1 to alcoholism and anxiety; 1 to atrial fibrillation and cardiovascular accident; 1 to alcoholism: 1 to posttraumatic stress disorder and hallucinations; and 1 to attention deficit hyperactivity disorder, anxiety and depression) and 6 HC participants (4 to relevant RBD symptomatology according to RBDSQ, 1 to seizure, 1 to cardiac arrythmia and cardiac problems).
